# Differentiation Model Establishment and Differentiation-Related Protein Screening in Primary Cultured Human Sebocytes

**DOI:** 10.1155/2018/7174561

**Published:** 2018-04-05

**Authors:** Man-Feng Zhang, Xiao-Lin Cai, Kai-Peng Jing, Xiao-Xue Pi, Pei-Yu Liao, Shi-Jie Li, Wen-Li, Chuan-Chuan Cai, Juan-Hua Quan, Yi-Ming Fan, Ge-Shi

**Affiliations:** ^1^Department of Dermatology, Affiliated Hospital of Guangdong Medical University, Zhanjiang, Guangdong, China; ^2^Department of Nephrology, Affiliated Hospital of Guangdong Medical University, Zhanjiang, Guangdong, China; ^3^Department of Gastroenterology, Affiliated Hospital of Guangdong Medical University, Zhanjiang, China

## Abstract

Sebocyte differentiation is a continuous process, but its potential molecular mechanism remains unclear. We aimed to establish a novel sebocyte differentiation model using human primary sebocytes and to identify the expression profiles of differentiation-associated proteins. Primary human sebocytes were cultured on Sebomed medium supplemented with 2% serum for 7 days. Flow cytometry showed that S phase cells were decreased time-dependently, while G1 and subG1 (apoptosis) phase cells increased under serum starvation. Transmission electron microscopy and Oil Red O staining revealed a gradual increase of intracellular lipid accumulation. Expression of proliferation marker was diminished, while expression of differentiation, apoptosis, and lipogenic markers elevated gradually during 7-day culture. iTRAQ analysis identified 3582 expressed proteins in this differentiation model. Compared with day 0, number of differentially expressed proteins was 132, 54, 321, and 96 at days 1, 3, 5, and 7, respectively. Two overexpressed proteins (S100 calcium binding protein P and ferredoxin reductase) and 2 downexpressed proteins (adenosine deaminase and keratin 10) were further confirmed by Western blot and immunohistochemistry.

## 1. Introduction

Sebocytes can produce lipid-rich sebum to function in epidermal barrier, hair follicle integrity, and antibacterial and antioxidant properties [1, 2]. Abnormal sebum secretion is involved in some common dermatosis including acne vulgaris, atopic dermatitis, psoriasis, rosacea, and seborrheic dermatitis [3, 4]. Sebocyte differentiation in mice is divided into undifferentiated (stem and proliferating cells) and differentiated (maturating and fully differentiated cells) stages that are, respectively, characterized by keratin 5 (K5) and peroxisome proliferator-activated receptor *γ* (PPAR*γ*) expression [5]. The terminal differentiation of human sebocytes presents with increased cell size, cytoplasmic accumulation of lipid droplets, and nuclear degeneration, followed by holocrine secretion and cell death [6, 7]. A recent study of mouse model unraveled that holocrine secretion of sebum is a DNase2-mediated form of programmed cell death distinct from apoptosis, necroptosis, and cornification [8]. Zouboulis et al. firstly compared the markers of human sebocyte versus keratinocyte differentiation in vitro in 1991 [9]. Cell confluence levels generally serve as differentiation stages of in vitro cultured sebocytes [10, 11]. However, to date a well-established differentiation model of human primary sebocytes has been unavailable, and the feasible markers of sebocyte differentiation have not been fully elucidated, which may hinder to understand the physiological function of sebaceous glands and pathophysiological mechanisms of sebaceous gland-related diseases.

PPAR*γ*, liver X receptors (LXR), sterol regulatory element binging protein (SREBP1), and Forkhead box O1 (FoxO1) may be main lipogenic factors for sebocytes [12–15]. PPAR*γ* could be a potential marker of sebocyte differentiation because its expression correlates with the differentiation stage of sebocytes [12]. LXR agonists inhibited proliferation but promoted lipogenesis in SZ95 sebocytes [13]. SREBP1 may be a key regulator of lipid synthesis in sebaceous glands through inducing expression of lipogenic genes [14, 16]. FoxO1 might be implicated in acne pathogenesis by mediating androgen receptor, PPAR*γ*, LXR, and SREBP1 expression [15, 17, 18]. Additionally, our previous study showed that sex determining region Y-related high mobility group box 9 (Sox9) can promote sebocyte proliferation, differentiation, and lipogenesis [10]. However, expression levels of these lipogenic factors at diverse differentiation stages are undetermined. In this study, we first established a differentiation model of human primary sebocytes. In addition, the differentially expressed proteins were identified by iTRAQ-based quantitative proteomic analysis, and 4 candidate proteins were further validated.

## 2. Materials and Methods 

### 2.1. Ethics Statement

The normal scalp for sebocyte isolation and culture was obtained from 5 patients undergoing plastic surgery, while facial skin for immunohistochemistry was taken from 3 patients with acne vulgaris and 3 healthy subjects. This study was approved by the ethical committee of our hospital (number PJ2012055). The written informed consent was obtained from all participants.

### 2.2. Cell Culture and Differentiation Model Construction

Primary sebocyte cultures were performed according to a previously described method [10, 11]. Briefly, the isolated sebaceous glands from the separated epidermis of normal scalp were transferred to a tissue culture dish. The isolated cells were cultivated in Sebomed basal medium (Biochrom GmbH, Berlin, Germany) containing 10% fetal bovine serum (FBS; Gibco BRL, Rockville, MD, USA) and 5 ng/ml recombinant human epidermal growth factor (Invitrogen) in a 5% CO_2_ incubator at 37°C. The cells were harvested with 0.05% Trypsin-EDTA (Gibco BRL) and subcultured after they became subconfluent. The cells after the second passage were used in this study. Sebocytes grown to 50% confluence were serum starved for 24 h in Sebomed without FBS, and the cells at this time point were designated as day 0 (D0). To induce differentiation, the D0 sebocytes were switched into Sebomed supplemented with 2% FBS and cultured up to 7 days with medium change every day.

### 2.3. Transmission Electron Microscopy

Sebocytes plated onto Aclar discs were induced to differentiate for various times and then fixed in 2.5% glutaraldehyde at 4°C overnight, followed by postfixation in 1% osmium tetroxide for 1 h. Samples were dehydrated and embedded in EPON-812 resin (TAAB Laboratories, Berkshire, UK). Ultrathin sections (70 nm) were cut with Leica EM UC7 ultramicrotome (Leica Microsystems, Wetzlar, Germany), doubly stained with uranyl acetate and lead citrate, and observed with JEM-1400 transmission electron microscope (JEOL, Tokyo, Japan) at 80 kV.

### 2.4. Oil Red O Staining

Sebocytes grown onto a 12-well culture plate were either left untreated or incubated in Sebomed with 2% FBS to induce differentiation. Intracellular lipid level was measured using Oil Red O staining as described by Li et al. [11].

### 2.5. Cell Cycle Distribution and Apoptosis Determination

Cell cycle and apoptosis were determined using Cell Cycle and Apoptosis Analysis Kits (Yeasen Corporation, Shanghai, China) according to the manufacturer's instructions. Briefly, after preparation of single-cell suspension, the cells were subjected to FACS Canto II flow cytometer (BD Biosciences, San Jose, CA, USA), and cell populations within G1, S, G2, and SubG1 (apoptosis) phages were measured using ModFit LT 3.2 software.

### 2.6. Western Blot

Cells were collected and lysed in Proprep solution (Intron, Daejeon, Korea). The concentration of obtained protein was measured using Bradford protein assay kit (Bio-Rad Laboratories, Hercules, CA, USA). Samples were then run on SDS-polyacrylamide gels, transferred onto nitrocellulose membranes, and incubated with following primary antibodies: Sox9, SREBP1, P53, P21, ADA, K5, K10, S100P, and actin (Santa Cruz Biotechnology, Santa Cruz, CA, USA); FoxO1, PPAR*γ*, and LXR (Cell Signaling Technology, Danvers, MA, USA); and FDXR (Abcam, Cambridge, MA, USA). Blots were incubated with peroxidase-conjugated secondary antibodies (Life Technologies) and visualized using enhanced chemiluminescence (Intron).

### 2.7. iTRAQ Coupled Liquid Chromatography-Tandem Mass Spectrometry (LC-MS/MS) Analysis

Protein samples were prepared from sebocytes at D0, D1, D3, D5, and D7 and labeled using iTRAQ Reagent-8 plex multiplex kit (AB SCIEX, Foster City, CA, USA) according to manufacturer's instructions. The iTRAQ-labeled peptides were then subjected to high-pH reversed-phase fractionation in Agilent 1100 HPLC System (Agilent, Palo Alto, CA, USA) equipped with a Gemini-NX (Phenomenex, 00F-4453-E0, Torrance, CA, USA) column (4.6 × 150 mm, 3 *μ*m, 110 Å) as described elsewhere [19, 20]. The collected fractions were then examined by Orbitrap Elite mass spectrometer (Thermo Fisher Scientific, San Jose, CA, USA). The top ten most intense signals in the acquired MS spectra were selected for further MS/MS analysis. The detected protein threshold was set to achieve a 1% false discovery rate. Original data of mass spectrometry were searched through Mascot 2.2 (Matrix Science Limited, London, UK) under the software platform Proteome Discoverer 2.1 (Thermo Fisher Scientific) against UniProt human protein database (Uniprot-human-160524.fasta) [21, 22].

### 2.8. Immunohistochemistry

Immunohistochemistry was performed on the paraffin-embedded specimens according to a previously described method [10]. The primary antibodies included polyclonal mouse anti-human S100P (clone B10, 1 : 100 dilution; Santa Cruz), ADA (clone D10, 1 : 100 dilution; Santa Cruz), K10 (clone LH1, 1 : 100 dilution; Santa Cruz), and rabbit anti-human FDXR (ab122900, 1 : 100 dilution; Abcam).

All experiments were repeated at least three times with different batches of cells. Data were analyzed using Student's* t*-test. Statistical significance was set at *P* < 0.05.

## 3. Results

### 3.1. Establishment of Sebocyte Differentiation Model

**Figure 1:**
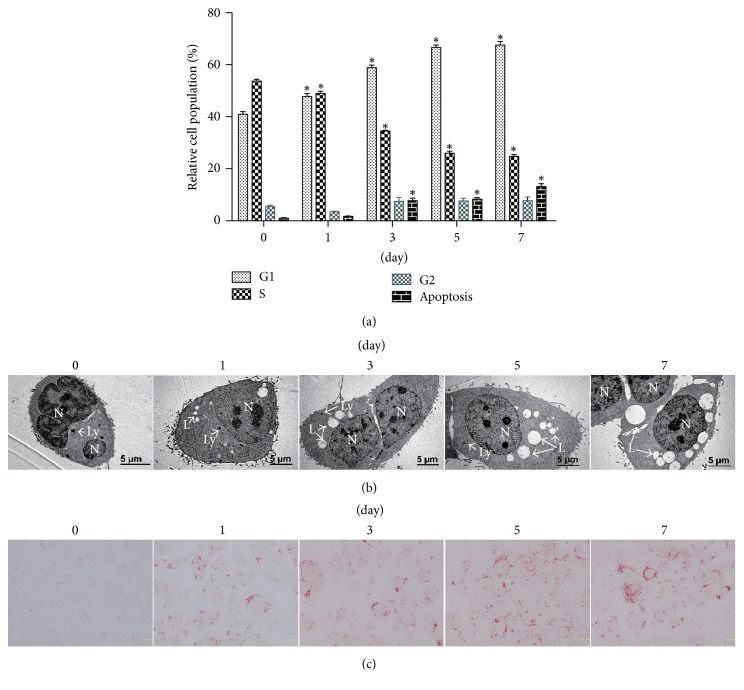
Establishment of human sebocyte differentiation model in vitro. Primary sebocytes cultured in growth media with 10% FBS were switched into media containing low serum concentrations (2% FBS) and incubated for 0, 1, 3, 5, and 7 days, respectively. (a) Cell cycle and apoptosis were determined using flow cytometry. (b) Transmission electron microscopy showed incremental number and volume of intracytoplasmic lipid vacuoles over a 7-day culture period (scale bars = 5 *μ*m). L, lipid vacuole; N, nucleus; Ly, lysosome. (c) Oil Red O staining exhibited a gradual increase of intracellular lipid accumulation (scale bars = 20 *μ*m). ^*∗*^*P* < 0.05, compared with day 0.

Sebocyte differentiation is a continuous process [23], but its potential molecular mechanism remains unclear. The accumulation of cytoplasmic lipid vesicles (lipid droplets) is a main feature of sebocyte differentiation [4]. Since the immortalized sebocyte lines only underwent partial differentiation [23], we used the primary human sebocytes to construct the in vitro differentiation model. Serum deprivation can induce G0/G1 cell cycle arrest, differentiation, and apoptosis in various cells [24]. The addition of 2% FCS induced expression of late differentiation markers in keratinocytes [25]. An 84 h culture led to 95% confluence rate in human sebocytes under 10% FBS [10]. Accordingly, we speculated that serum deprivation may also initiate sebocyte differentiation. To testify this hypothesis, human primary sebocytes were switched from growth media supplemented with 10% FBS into a basal medium containing 2% FBS, followed by simultaneous determination of cellular DNA contents and apoptosis using flow cytometry. The percentages of S phase cells were decreased time-dependently by serum starvation, while those of G1 and subG1 (apoptosis) phases increased (Figure 1(a)). In addition, transmission electron microscopy revealed that small lipid vacuoles were present in the cytoplasm at day 1, and their number and volume were gradually incremental thereafter over a 7-day culture period (Figure 1(b)). Oil Red O staining showed a gradual increase of intracellular lipid accumulation (Figure 1(c)). Together, these findings suggest that serum deprivation can effectively trigger differentiation of primary sebocytes.

### 3.2. Detection of Lipogenic, Proliferation, Differentiation, and Apoptosis Markers in Sebocyte Differentiation Model

**Figure 2:**
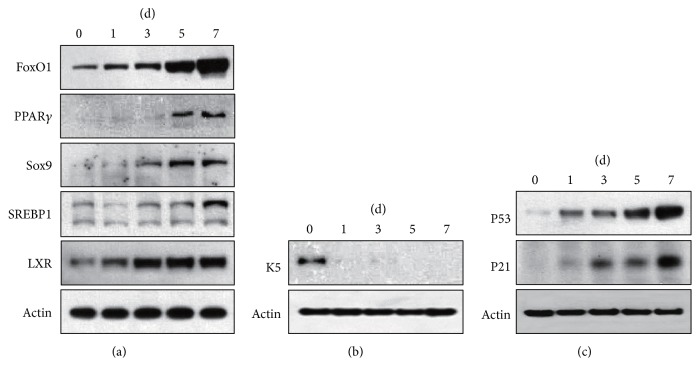
Expression of proliferation, differentiation, apoptosis, and lipogenic markers in sebocyte differentiation model. Primary sebocytes were incubated for 0, 1, 3, 5, and 7 days under serum deprivation. The cells were then lysed and subjected to immunoblotting analysis using antibodies against FoxO1, Sox9, PPAR*γ*, SREBP1, and LXR (a) or K5 (b) or P53 and P21 (c). Actin was used as a loading control.

To validate the sebocyte differentiation model, we firstly examined expression of lipogenic factors by Western blot. FoxO1, LXR, Sox9, and SREBP1 levels were increased time-dependently, with a top at D5 and D7 (Figure 2(a)). Then, expressions of proliferation, differentiation, and apoptosis markers were detected. We found that K5 was prominent (Figure 2(b)) at D0, while PPAR*γ* was remarkable at D5–D7 (Figure 2(a)). P53 and P21 were almost undetectable at D0 but progressively increased after D1 (Figure 2(c)). These data support the successful establishment of human sebocyte differentiation model and imply that the sebocytes at D0 and at D5–D7, respectively, represent undifferentiated and fully differentiated cells.

### 3.3. Identification of Differentially Expressed Proteins in Sebocyte Differentiation Model

**Figure 3:**
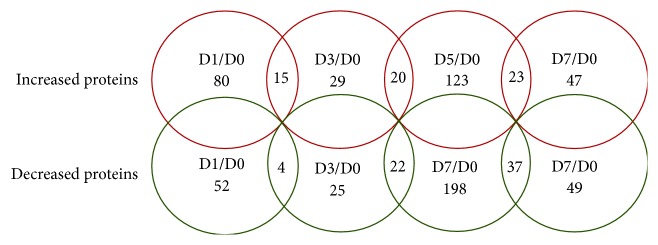
Proteome alterations during human sebocyte differentiation in vitro. Human primary sebocytes were induced to differentiate by serum deprivation. These cells were harvested at days 0, 1, 3, 5, and 7 and then subjected to the iTRAQ-based quantitative proteomic analysis. Numbers of up- and downregulated proteins in differentiated sebocytes at days 1, 3, 5, and 7 were shown. The expression profile of cells at day 0 was used as a control.

To gain further insights into the mechanisms of sebocyte maturation, the differentially expressed proteins in undifferentiated sebocytes (D0), maturating sebocytes (D1 and D3), and fully differentiated sebocytes (D5 and D7) were identified using an iTRAQ-based quantitative proteomic approach. The differentially expressed proteins were screened using a fold change cut-off of ±1.5. A total of 3582 proteins were identified from 17939 peptides during sebocyte differentiation, which were matched with 205655 MS/MS spectra (data not shown). Compared with D0, the number of differentially expressed proteins was 132 at D1, 54 at D3, 321 at D5, and 96 at D7 (Figure 3; Table S1). Based on the literature, the skin-associated functions of 41 differentially expressed proteins at D7 in human skin are summarized in Table 1.

### 3.4. Validation of 4 Candidate Proteins during Sebocyte Differentiation

**Figure 4:**
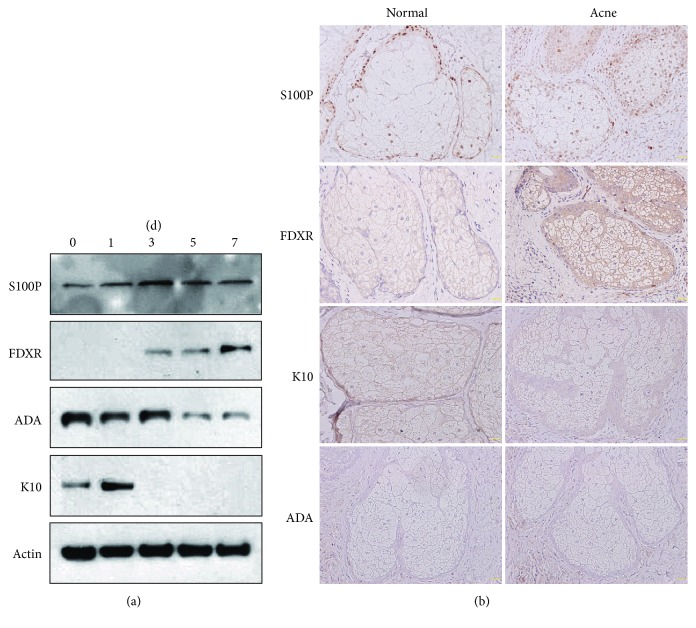
Expression of 4 candidate proteins in sebocytes and acne lesion. (a) Human primary sebocytes were cultivated under serum deprivation for indicated times, and expression levels of S100P, FDXR, ADA, and K10 were monitored by Western blot. Actin was used as a loading control. (b) Representative immunostaining images of S100P, FDXR, ADA, and K10 in sebaceous glands of acne lesion and normal skin (scale bars = 50 *μ*m).

To further confirm the iTRAQ results, we chose 4 significantly differentially expressed proteins that may be related to proliferation, differentiation, and apoptosis in sebocytes or keratinocytes, of which S100 calcium binding protein P (S100P) and ferredoxin reductase (FDXR) were upregulated but adenosine deaminase (ADA) and K10 were downregulated (Table 1). Western blot showed that expression of S100P and FDXR was remarkably increased either in maturing sebocytes or in fully differentiated sebocytes compared to undifferentiated cells, while expression of ADA and K10 was reverse (Figure 4(a)). Furthermore, immunostaining of these proteins was performed on acne lesion and normal skin. K10 was located in the cytoplasm, S100P in the nucleus, and FDXR in the cytoplasm and nucleus of sebocytes. Compared with normal skin, S100P and FDXR expression were significantly higher but K10 expression was slightly lower in acne lesion. However, ADA immunoreactivity was negative in both acne lesion and normal skin (Figure 4(b)). These results suggest that S100P, FDXR, and K10 may regulate human sebocyte differentiation and contribute to acne pathogenesis.

## 4. Discussion

Both sebocytes and keratinocytes seem to originate from bipotential progenitor stem cells [26], but their differentiation fates are different. Keratinocyte differentiation has been explored in numerous cell models [25], but less is known about sebocyte differentiation. Differentiation may be induced by various procedures. High Ca^2+^, low serum, confluence, and lower incubation temperature can influence keratinocyte differentiation [25]. Nevertheless, low concentration of extracellular Ca^2+^ may enhance sebocyte differentiation [26]. The incremental cell density in continuous cultures is a common method to induce sebocyte differentiation in vitro [9–11]. Sebocyte confluence levels approached 20%, 50%, 75%, and 95% when cultivated on the medium containing 10% FBS for 12 h, 36 h, 60 h, and 84 h, respectively [10]. Serum deprivation induces differentiation, apoptosis, and G0/G1 cell cycle arrest in various cells [24]. The 2% FCS can more efficiently trigger expression of late differentiation markers than high Ca^2+^ switch in keratinocytes [25]. Thus, we decided to reduce the serum concentration from 10% to 2%, with attempts to slow down proliferation and extend differentiation process. Ultrastructural observation and Oil Red O staining uncovered that intracellular lipid accumulation was time-dependently increased during 7-day culture period. Flow cytometry revealed a time-dependent decrease in S phase and increase in G1 and subG1 (apoptosis) phases. Our results suggest serum deprivation can be used to construct the human sebocyte differentiation model.

In order to validate the reliability of sebocyte differentiation model, proliferation, differentiation, apoptosis, and lipogenic markers were determined. K5 and PPAR*γ* can act as proliferation and differentiation markers in mouse sebocytes, respectively [5]. P53 can suppress cell cycle and enhance apoptosis, and its target gene P21 is required for the apoptotic action [27]. LXR, Sox9, FoxO1, and SREBP1 seem to be the main lipogenic factors for sebocytes [10, 13–15]. In this sebocyte differentiation model, K5 was only expressed at D0, while PPAR*γ* was significantly detectable at D5–D7. Expression levels of P53, P21, LXR, Sox9, FoxO1, and SREBP1 increased progressively after D1. Based on these findings, we propose that the sebocytes at D0, D1–D3, and D5–D7 in this differentiation model might represent proliferative, early differentiated, and fully differentiated cells, respectively.

Quantitative proteome analysis has not been performed during sebocyte differentiation process. In our sebocyte differentiation model, numbers of differentially expressed proteins reached the top at D5 and the nadir at D3 compared with D0, suggesting that sebocyte differentiation may be the most remarkable at D5. Intriguingly, except that P53 expression was 1.2-fold only at D7, expression of the differentiation and lipogenic markers was absent from D0 to D7. Aebersold et al. proposed that the quality of MS data is superior to that of Western blot [28]. It is unclear whether the iTRAQ results may represent the general view of this sebocyte differentiation model, and these data remain to be further testified using targeted proteomic methods.

Of 41 skin-associated proteins at D7, 2 upregulated (FDXR, S100P) and 2 downregulated (ADA and K10) proteins were selected, and their expression in vitro and in vivo was detected by Western blot and immunohistochemistry. FDXR, a mitochondrial enzyme that catalyzes the reduction of ferredoxin, may regulate epidermal differentiation via elevating reactive oxygen species [29]. S100 proteins belong to the superfamily of calcium binding proteins and implicate in the regulation of many cellular processes including growth, differentiation, cycle progression, transcription, and secretion [30]. Although ADA is mainly involved in immune system development in humans, it is also critical for cell proliferation and differentiation. The p63 gene knockdown can reduce proliferation and ADA expression in human keratinocytes [31]. K10 is the common keratinization-associated keratin of suprabasal keratinocytes in the epidermis [32]. The targeted deletion of K10 reinforced sebocyte proliferation and differentiation in mice [33]. Western blot results displayed overexpression of S100P and FDXR and downexpression of ADA and K10 in differentiated sebocytes, which testifies the iTRAQ analysis. In addition, immunostaining demonstrated overexpression of S100P and FDXR but mild downexpression of K10 in acne lesion, while ADA expression was absent in both acne lesion and normal skin. These results imply that S100P and FDXR may enhance but K10 and ADA reduce human sebocyte differentiation, and S100P, FDXR, and K10 could be involved in the acne pathogenesis. However, further studies are needed to ascertain their molecular mechanisms on sebocyte proliferation and differentiation.

In conclusion, a human sebocyte differentiation model is successfully established using serum deprivation. Meanwhile, iTRAQ quantitative proteomics analysis demonstrates presence of 96 differentially expressed proteins at D7, with 41 proteins possessing skin-associated functions. This in vitro differentiation model of human sebocytes may be helpful to investigate the regulatory mechanism of sebaceous gland and pathogenesis of sebaceous gland-associated disorders. Nevertheless, the roles of these differentially expressed proteins in sebocyte differentiation await further studies.

## Figures and Tables

**Table 1:** Skin-associated functions of 41 differentially expressed proteins at D7 compared with D0.

Number	Uniprot accession	Protein name	Skin-associated functions	Fold change
(1)	P20591	MX dynamin like GTPase 1	Epidermal defense against viruses	2.9
(2)	P11117	Acid phosphatase 2	Skin homeostasis	2.5
(3)	Q03405	Plasminogen activator, urokinase receptor	Migration of epidermal keratinocytes and outer root sheath cells during human morphogenesis	2.2
(4)	P25815	S100 calcium binding protein P^*∗*^	Tumor growth, invasion and metastasis	2
(5)	Q460N5	Poly(ADP-ribose) polymerase family member 14	Cytokine production in allergic inflammation models	1.8
(6)	Q7Z4W1	Dicarbonyl and L-xylulose reductase	Cell adhesion in normal keratinocytes, melanocytes and endothelial cells	1.8
(7)	Q9NVV5	Androgen induced 1	Involved in androgen-regulated hair cycle	1.8
(8)	P27701	CD82 molecule	Suppress melanoma cell migration and invasion	1.8
(9)	A0A024RB23	Diacylglycerol kinase alpha	Diacylglycerol-mediated lipid homeostasis and profibrotic fibroblast activation	1.8
(10)	O95471	Claudin 7	Epidermal neoplastic process	1.7
(11)	Q53FA7	Tumor protein p53 inducible protein 3	P53-regulated proapoptosis	1.7
(12)	X6R8F3	Lipocalin 2	Regulate growth, differentiation and migration of squamous cell carcinoma	1.7
(13)	Q9Y3Z3	SAM and HD domain containing deoxynucleoside triphosphate triphosphohydrolase 1	Limit HIV-1 infection in macrophages and regulate cell cycle progression in fibroblasts	1.6
(14)	B0QYD3	Apolipoprotein B mRNA editing enzyme catalytic subunit 3B	Induce somatic mutations and involve in pathomechanism of several cancers (melanoma)	1.6
(15)	Q00978	Interferon regulatory factor 9	Inflammation	1.6
(16)	P05161	ISG15 ubiquitin-like modifier	Regulate expression of pro-inflammatory chemokines and cytokines	1.6
(17)	P82673	Mitochondrial ribosomal protein S35	Activate inflammatory signaling	1.6
(18)	P09914	Interferon induced protein with tetratricopeptide repeats 1	Immunomodulation and interferon response	1.6
(19)	E7ESA6	Protein tyrosine kinase 2	Keratinocyte migration	1.6
(20)	A0A0A0MT64	Ferredoxin reductase^*∗*^	Epidermal differentiation	1.6
(21)	A0A024R9E4	Mal, T-cell differentiation protein 2	May induce malignant mouse skin squamous cell carcinomas	1.6
(22)	P13645	Keratin 10^*∗*^	Keratinization-associated keratin, sebocyte proliferation and differentiation	−3.8
(23)	P31151	S100 calcium binding protein A7	Epithelial inflammation	−2.5
(24)	Q9Y5K6	CD2 associated protein	Expression of plasmacytoid dendritic cells in dermis	−2.4
(25)	P08240	SRP receptor alpha subunit	Regulate keratinocyte proliferation by affecting cell cycle progression	−2.2
(26)	P08779	Keratin 16	Associated with epidermal hyperproliferation	−1.9
(27)	P02533	Keratin 14	Major keratin of basal keratinocytes	−1.9
(28)	P29034	S100 calcium binding protein A2	Keratinocyte differentiation and carcinogenesis	−1.8
(29)	P02760	Alpha-1-microglobulin/bikunin precursor	Related to apoptosis or antiproliferation and might have causal roles in psoriasis	−1.7
(30)	Q9NS00	Core 1 synthase, glycoprotein-N-acetylgalactosamine 3-beta-galactosyltransferase 1	Immune responses of CD8+ T cells and skin allograft survival	−1.7
(31)	P35908	Keratin 2	Keratinocyte differentiation	−1.7
(32)	Q15582	Transforming growth factor beta 1	Promote proliferation in melanoma cells	−1.7
(33)	P04040	Catalase	Oxidative stress causes abnormal keratinocyte proliferation and differentiation	−1.7
(34)	P02649	Apolipoprotein E	Epidermal proliferation and differentiation	−1.6
(35)	P04259	Keratin 6B	Markers of epithelial cell differentiation	−1.6
(36)	Q9Y4K0	Lysyl oxidase like 2	Inhibit keratinocyte differentiation, promote development of squamous cell carcinoma	−1.6
(37)	P18074	ERCC excision repair 2, TFIIH core complex helicase subunit	Important DNA repair molecule for initiating cutaneous melanoma	−1.6
(38)	A0A0S2Z381	Adenosine deaminase^*∗*^	Immune system development, cell proliferation and differentiation	−1.6
(39)	Q92626	Peroxidasin	Basement membrane integrity and homeostasis	−1.6
(40)	Q14117	Dihydropyrimidinase	Enriched in somatic mutations of melanoma	−1.6
(41)	Q05209	Protein tyrosine phosphatase, non-receptor type 12	Related to invasiveness of melanoma cells	−1.6

^*∗*^Four proteins have been further validated in this study.

## Abbreviations

ADA: Adenosine deaminase
AR: Androgen receptor
FDXR: Ferredoxin reductase
FoxO1: Forkhead box O1
iTRAQ: Isobaric tags for relative and absolute quantitation
K5: Keratin 5
K10: Keratin 10
LXR: Liver X receptors
PBS: Phosphate-buffered saline
PPAR*γ*: Peroxisome proliferator-activated receptor *γ*
S100P: S100 calcium binding protein P
SGs: Sebaceous glands
Sox: Sex determining region Y-related high mobility group box
SREBP: Sterol regulatory element binging protein.

## Supplementary Materials

Supplementary Materials Table  S1: number of differentially expressed proteins at D1, D3, D5, and D7 compared with D0 in human sebocyte differentiation model.
