# Supplementary material for: Differentiation Model Establishment and Differentiation-Related Protein Screening in Primary Cultured Human Sebocytes
Source: Biomed Res Int. 2018 Apr 5;2018:7174561. doi: 10.1155/2018/7174561 (PMC5907408; doi:10.1155/2018/7174561)
Supplement: Supplementary Materials — Table S1: number of differentially expressed proteins at D1, D3, D5, and D7 compared with D0 in human sebocyte differentiation model. [file 7174561.f1.docx]

Table S1 Number of differentially expressed proteins at D1, D3, D5, and D7 compared with D0 in human sebocyte differentiation model

|  | UniProt accession number |
| --- | --- |
| D1/D0 |  |
| Increased proteins (*n*=80) | A0A0J9YXX4, H6VRG1, P02533, P08779, P35908, Q24JP5, A0A0F7KYT8, Q15031, P11117, Q9BUB7, P04259, P62891, A0A024R0C0, Q9HAF1, O95817, J3QR44, P98198, Q5TG30, P15291, H6VRG2, Q6DN03, A0A024R7E0, Q96LA8, Q9H8H0, P13995, Q9NQG5, B7ZKM6, Q9NVU7, A0A024R2T0, P36955, Q7KZN9, O00217, A0A024R396, A0A0A0MSI8, E7ERK9, A0A087WT20, P13645, P11802, Q6P2Q0, A4D0P7, Q7LBC6, Q8WVY7, Q96T51, Q08379, Q13885, A0A024R648, Q8WUA2, A0A0U1RRH6, Q6UW78, A0A024R050, A0A024R329, Q15813, Q9NRX2, Q9H6K4, P62861, Q6IA86, G3V5Z7, A6QKW0, Q53FA7, Q8WZ82, P12532, Q92997, Q16512, Q8IVM0, Q9Y2S7, Q8NFV4, Q8IXM3, Q96GM8, Q9H0R4, O95471, Q9UHR4, Q9NYL2, Q562L9, A0A0D9SF58, Q96D53, J3KMX3, A6NHL2, P22830, Q86YZ3, P35527 |
| Decreased proteins (*n*=52) | O75330, Q6IBU4, Q9UG56, Q5TEJ8, Q5U091, Q96GC5, P29034, A6NMH8, Q969S9, Q7L5N1, E7EW49, P00734, H0UI80, A0A087WXI7, K7ELG9, P49815, Q9H147, V9HW44, Q99543, Q496C9, Q9UEW8, Q8NHQ9, P62312, P01040, Q15397, Q96GI7, J3QL71, P09132, D3YTG3, Q86X10, P22223, Q96RN5, G5EA09, P60866, Q8N0U4, Q9NQT5, A0A024R7N7, J3KMZ8, B2R4M6, P35558, Q9Y5K6, X6R8F3, G9FP35, A8K070, A0A024R3X1, Q96EK5, P04350, Q9GZZ9, Q5TDH0, O60341, P31151, A0A024R5L0 |
| D3/D0 |  |
| Increased proteins (*n*=29) | Q9HAF1, A4D0P7, A0A024R050, P11117, J3KMX3, A0A024R3E3, O95817, Q6P2Q0, Q8WUA2, A0A024R035, Q9NU22, Q14533, Q15828, Q9BUB7, D6RF35, O00217, B0QYD3, D3DRR6, A0A0U1RRH6, P49459, A0A024R0C0, Q24JP5, B2RWN5, Q03405, J3QR44, F5GXV7, I7JB59, P41223, P62891 |
| Decreased proteins (*n*=25) | Q9Y2J4, Q9BXW9, P61803, A0A0C4DH83, Q8TE01, B5MCP4, Q9Y4K0, P35908, Q15031, Q14117, H6VRG1, L0R6G9, A0A126GW78, B7ZLJ0, Q9P219, O95758, Q9Y623, P13640, Q5TDH0, A0A024R4Z9, P01040, Q969S9, Q9UKX2, P13645, P31151 |
| D5/D0 |  |
| Increased proteins (*n*=123) | A0A059QFD5, P11117, Q9NU22, Q03405, A0A0U1RRH6, O00217, Q9UQ13, A4D0P7, A6NHL2, F5GXV7, Q68E01, A0A0F7G8J1, B4E1Z4, P62861, O75911, O95260, P62891, A0A024R7E0, J3KMX3, Q9NUI1, Q96D53, Q9H8H2, J3QR44, Q8NFZ8, A0A024R3E3, D6RF35, P36955, A0A024R050, A8K7S5, Q8WUA2, A0A024R0C0, Q6P2Q0, A0A0K0K1H8, O75942, Q14691, A0A024R075, A0A087WWB6, B7ZKQ8, P08697, A0A087WYN9, A0A024RB23, P35914, O43815, O95071, Q99880, Q96EK4, A0A0D9SEY1, Q9NVU7, A0A024R944, Q8WUK0, Q96HR3, Q9P1F3, Q5SSJ5, Q9BWU0, O75843, Q9NX24, Q96A59, P15529, Q96RU3, Q9NXV6, Q96RT1, Q86X29, Q9NYZ3, I7JB59, B4DPY1, Q5C9Z4, Q9NRX2, Q5TG30, Q9Y618, Q96A73, A0A0D9SF58, Q9NVV5, J3QK89, Q13325, Q96GM8, Q9NVP2, Q13469, P46776, Q8N2F6, O15066, A0A024R9L1, Q8NC96, P18031, D3DRR6, Q7Z7H8, Q6IBU4, Q6NUQ4, Q15628, P08582, Q86TB9, Q9H6K4, B7Z1P2, Q08623, A6NHR9, A0A024R035, A6NFI3, Q96CN7, P09417, Q9NQG5, Q8WVJ2, Q6FG99, K7ELC2, Q5VTI5, Q5SRE5, E7ESA6, A0A087WXI5, P25815, A0A024R3M7, P82675, Q16512, Q8IUH3, Q9NZN4, P02753, Q53FA7, O95817, Q8WUW1, P62136, Q15257, P98198, J3KPP4, A4ZVS7, P50897, O00622 |
| Decreased proteins (*n*=198) | O15116, G3XAI2, Q96EB6, A0A024R5Y1, Q9BZJ0, Q9NX18, P49753, A0A024RC42, Q9BST9, Q8NBU5, Q9ULH1, A0A024R371, A0A024QZE1, P58546, P20585, A0A024QYW3, Q8N543, L0R4W3, Q14690, P48775, Q13868, Q9NUY8, A0AV96, A7XZE4, O00308, O43156, A0A024R5L0, O94927, A0A024RDQ8, A0A0D9SFE4, Q9Y2W2, O94919, Q8NB90, Q6IBS0, O75489, Q6NVY1, Q86SX6, Q9NYB0, P00167, A0A0B4J1W0, Q13033, P53634, Q8TE01, O15231, A0A024R9G7, P04040, Q15582, Q15369, Q99615, A0A024RDJ1, Q2L6I0, H0UI06, P04259, Q9BRF8, Q9NQ92, Q15436, Q06203, Q2TAA2, Q9Y4X5, Q9P253, A4D1V4, P08779, Q9P0P0, G5EA09, Q14117, Q9Y623, Q15904, F1T0A5, Q7Z2W9, Q9NVM6, Q9UBW8, A0A024RAF1, Q9BXW9, Q53FV1, Q9P0U4, P08240, P26038, P62834, Q9H6R4, Q86X10, B7ZLJ0, A0A024R9T3, A0A0C4DGX5, Q92626, Q9Y4K0, Q8IY17, A0A024R8D2, A0A024R4Z9, A0A0A0MTQ8, Q9Y333, Q96HQ2, Q96EK5, Q7L2H7, A0A024RAM4, B2RXF3, O15551, O75146, Q8IVP5, P02649, Q01650, V9HW44, Q9UJF2, L0R6G9, P80723, Q7L523, O60248, P22830, Q15031, O60936, A0A087X256, O95486, Q9P219, Q02224, Q7Z7N9, Q9Y221, P14550, Q6ZRP7, Q969T4, P12109, Q9Y547, A0A126GW78, P82912, A0A0C4DH83, P55039, Q9UK33, Q9Y5S5, A0A024R895, A0A087WWM0, O00743, Q9NQT5, Q9NS00, A0A024R978, P60866, A7E2W2, B0YIW2, Q96PU5, Q9H8H0, Q6PCE3, P18074, B7ZKM6, Q9NSV4, Q6DN03, P29034, B7WNH4, Q9BY32, A0A087X2G6, Q9H8P0, Q9BW92, A0A024R8U1, A0A0A0MTL6, A0A024R683, A8K3S1, B1PS43, Q9H4B7, P20020, Q9GZL7, Q8IXI1, O15160, P02533, A0A0B4J2A0, O95758, Q8TC07, A0A024R625, A3F768, Q96K76, Q9Y5K6, Q9BY08, Q8WW12, Q92552, O60645, Q4G0J3, Q9UBC2, Q5TEJ8, Q9GZP4, Q8IUR7, A0A024QYX0, Q96GC5, Q9UI26, Q9H147, Q8N0U4, H6VRG1, E9PAU2, P01040, Q9H3Z4, P62072, Q9Y2J4, P61803, Q9Y3D6, B5MCP4, O60658, Q9P0L0, P49593, A0A024R172, P31151, Q496C9, P13645, Q9UKX2, Q5TDH0 |
| D7/D0 |  |
| Increased proteins (*n*=47) | P20591, O75911, P11117, A0A024R050, Q8WUA2, Q03405, A0A024R0C0, Q9BUB7, Q5U0D2, P62891, Q24JP5, P25815, Q9HAF1, P07305, J3QR44, Q92520, Q9BYK8, Q460N5, Q7Z4W1, Q9NVV5, P27701, Q8N2F6, A0A024RB23, O95071, O95471, Q53FA7, P09417, Q7KZN9, Q86X29, A4D0P7, A0A024R2T0, X6R8F3, Q8TBF4, Q9Y3Z3, Q92508, P35914, Q9H8H2, B0QYD3, O00217, P98198, Q00978, P05161, Q9NRX2, P82673, P09914, E7ESA6, A0A0A0MT64 |
| Decreased proteins (*n*=49) | P13645, Q5TDH0, Q9UKX2, Q86X10, Q96GC5, P31151, Q9Y5K6, P08240, A0A024R9D9, P01040, P08779, P02533, Q9P0P0, Q9P0L0, Q4G0J3, D9ZGG2, P29034, Q9UBC2, Q9BY32, O95758, P02760, Q9NS00, A0A0B4J1W0, P35908, Q15582, O60248, Q9H3Z4, P04040, Q9BXB4, P02649, Q9NYB0, P04259, Q9Y4K0, Q9Y3D3, P18074, Q7L2H7, A0A024QYX0, Q15031, Q9NX18, A0A0S2Z381, O15121, Q8IY17, Q92626, Q99638, Q14117, Q05209, Q9Y2W2, P04350, Q96B97 |

The differentially expressed proteins were listed in descending order of t
